# Metagenome-assembled genomes of two nitrifying microorganisms from the freshwater archaeal ammonia-oxidizing enrichment culture BO1

**DOI:** 10.1128/mra.00900-23

**Published:** 2024-01-24

**Authors:** Annette Bollmann, Christopher J. Sedlacek, Luis Sayavedra-Soto, Jeanette M. Norton

**Affiliations:** 1 Department of Microbiology, Miami University, Oxford, Ohio, USA; 2 Department of Botany and Plant Pathology, Oregon State University, Corvallis, Oregon, USA; 3 Department of Plants, Soil and Climate, Utah State University, Logan, Utah, USA; Montana State University, USA

**Keywords:** ammonia-oxidizing archaea, ammonia oxidation, nitrification, enrichment culture, nitrite-oxidizing bacteria

## Abstract

Two metagenome-assembled genomes (MAGs) were recovered from the ammonia-oxidizing enrichment culture BO1 obtained from the sediment of the freshwater reservoir Lake Burr Oak, Ohio, USA. High quality MAGs were assembled for the archaeal ammonia oxidizer *Nitrosarchaeum* sp. BO1 and the canonical nitrite oxidizer *Nitrospira* sp. BO1.

## ANNOUNCEMENT

Three groups of microorganisms can carry out ammonia oxidation: ammonia-oxidizing archaea (AOA), ammonia-oxidizing bacteria, and complete ammonia oxidizers (1). These microorganisms are found ubiquitously; however, cultivation and especially isolation are difficult due to slow growth rates and low biomass production (2, 3). Here, we present the metagenome of an AOA enrichment culture obtained from the sediment of the freshwater reservoir Lake Burr Oak (39°54′N, 82°06′W) and two metagenome-assembled genomes (MAGs) obtained from the metagenome (Table 1).

**TABLE 1:** Overview over metagenome and the MAGs of the nitrifying microorganisms from the AOA enrichment culture BO1

	Metagenome	Bin003	Bin004
No. of contigs	1,185	8	11
*N*_50_	139,171	394,906	544,602
GC content (%)	54.73	32.8	59.55
Size (bp)	14,220,367	1,525,366	3,110,956
Completeness (%)		99.51	94.94
Contamination (%)		0	2.73
Phylogenetic affiliation		*Archaea* *Thaumarchaeota* *Nitrosarchaeum* sp. BO1	*Nitrospira* *Nitrospira* sp. BO1

The enrichment culture was started in 2008 by inoculating sediment (0.5 g) into buffered mineral salts medium (MS) with 0.25 mM ammonium as the sole substrate and incubated at 27°C (2, 4). When all the ammonium was consumed, the culture was filtered (0.45 µm) and the filtrate with the AOA was used as inoculum. This selection step was repeated four to five times. Afterward, the culture was continuously cultivated in MS medium with 0.5 mM ammonium and transferred every 4–6 weeks to fresh medium.

After around 6 years of cultivation, the metagenome of the enrichment culture was sequenced. Cells were collected by centrifugation (22,000 × *g*, 4°C, 20 min), and the DNA was isolated following the JGI bacterial genomic DNA isolation (CTAB) protocol (http://my.jgi.doe.gov/general/protocols/JGI-Bacterial-DNAisolation-CTAB-Protocol-2012.pdf). Whole genome libraries were prepared using the Nextera XT library preparation kit following the manufacturer’s recommendation and sequenced on an Illumina 1.9 HiSeq (Illumina, San Diego, CA, USA) with 2 × 300 cycles. A paired-end library with 2,870,329,980 bp and 9,535,980 reads was obtained.

The metagenome was assembled in KBase using the following software packages in the default setting (5). The reads were quality controlled with Trimmomatic-v0.36; the metagenome was assembled with MEGAHIT-v1.2.9, the assembly was binned with CONCOCT-v1.1 and quality controlled with CheckM-v1.0.18. The metagenome and the high-quality MAGs [as defined by Bowers et al. (6)] belonging to the nitrifying community members were annotated with RASTtk-v1.073 in KBase. The metagenome was uploaded for further evaluation to the Integrated Microbial Genomes and Microbiomes system of the Department of Energy-Joint Genome Institute and annotated through their pipeline (7). The OrthoANI similarities between the MAGs and the closest cultured relative were calculated using the ANI calculator on the webpage ezbiocloud.net (8).

The *amoA*, *amoX*, *amoC*, *amoB*, and the recently described *amoY* and *amoZ* genes, as well as many other genes typical for archaeal ammonia oxidizers, were present in the MAG of *Nitrosarchaeum* sp. BO1 (bin003), indicating that the MAG represents the genome of an archaeal ammonia oxidizer (9–11). The closest cultured relative was determined in KBase based on 49 core genes as *Nitrosarchaeum koreensis* MY1 (ANI 93.1%), an archaeal ammonia oxidizer isolated from garden soil (12–14).

The MAG *Nitrospira* sp. BO1 (bin004) represented a canonical nitrite oxidizer from the phylum *Nitrospira*. The MAG contained the essential nitrite oxidoreductase genes (*nxrAB*) and other genes typical for these nitrite oxidizers including the cyanase (*cyn*) gene (15–17). The closest cultured relative was *Nitrospira lenta* (Average Nucleotide Identity, ANI: 76.44%), an NOB isolated from a wastewater treatment plant (17).

## Data Availability

All sequences are deposited at GenBank under BioProject no. PRJNA1014936. The assembly and binning are publicly available in KBase https://narrative.kbase.us/narrative/135585, and the annotated metagenome is available in the DOE-JGI-IMG database under the IMG genome ID 3300061095.
